# Post retraction citations in context: a case study

**DOI:** 10.1007/s11192-017-2242-0

**Published:** 2017-03-03

**Authors:** Judit Bar-Ilan, Gali Halevi

**Affiliations:** 1 Department of Information Science, Bar-Ilan University, Ramat Gan, Israel; 2 Icahn School of Medicine at Mount Sinai, New York, NY USA

**Keywords:** Retracted articles, Post retraction citations, Positive citations, Negative citations, Neutral citation

## Abstract

This study examines the nature of citations to articles that were retracted in 2014. Out of 987 retracted articles found in ScienceDirect, an Elsevier full text database, we selected all articles that received more than 10 citations between January 2015 and March 2016. Since the retraction year was known for only about 83% of the retracted articles, we chose to concentrate on recent citations, that for certain appeared after the cited paper was retracted. Overall, we analyzed 238 citing documents and identified the context of each citation as positive, negative or neutral. Our results show that the vast majority of citations to retracted articles are positive despite of the clear retraction notice on the publisher’s platform and regardless of the reason for retraction. Positive citations can be also seen to articles that were retracted due to ethical misconduct, data fabrication and false reports. In light of these results, we listed some recommendations for publishers that could potentially minimize the referral to retracted studies as valid.

## Background

Recent studies on retracted articles show that the number of retracted articles is increasing in relative measure to the overall growth in scientific publications (Cokol et al. 2008; Marcus and Oransky 2014). The major reasons for articles to be retracted are: misconduct and error (Fang et al. 2012; Steen 2011a). Peer review is supposed to guard from publishing fraudulent results, however sometimes mistakes or unethical conduct (plagiarism) cannot be identified during the review process. Thus, when misconduct or unethical behavior are noticed, sometimes by the community, the article is retracted at the request of the editor, the author, the employer or the publisher.

Although the act of retracting flawed articles helps purge the scientific literature of erroneous or unethical research, citations to such research after its been retracted, presents a real challenge to the integrity of the scientific endeavor. Continued citations, or post-retraction citations, of articles that were withdrawn especially due to plagiarism, data falsification or any other unethical practices interferes with the process of eliminating such studies from the literature and research overall.

Essentially, there are two major types of post-publication citations of retracted papers; citations that an article received prior to its retraction and the citations that it received post retraction and despite retraction notices (Unger and Couzin 2006; Campanario 2000). Both types of citations put the scientific process in jeopardy, especially when they are cited as legitimate references to previous work and the reason for retraction was manipulation and fraud. Some studies have shown that retracted articles that received a high number of citations pre-retraction are more likely to receive additional citations post-retraction (Campanario 2000; Redman et al. 2008). One of the early studies on post retraction citations (Kochan and Budd 1992) examined post retraction citations to papers of John Darsee, and showed that over 85% of the post retraction citations are positive, not mentioning fraud or retraction. Other early studies include works by Pfeifer and Snodgrass (1990) and by Garfield and Welljams-Dorof (1990).

A more recent example is described in a study by Bornemann-Cimenti et al. (2015) who studied the case of Scott S. Reuben who was convicted of fabricating data in 25 of his studies which resulted in mass retractions of his articles. The authors of the study have shown that the popularity of Reuben’s articles did not diminish post-retraction even five years after the retractions. Another phenomenon identified in the literature is of authors’ self-citing their retracted articles and thus contributing to the perception that their retracted work is valid (Madlock-Brown and Eichmann 2015).

Other studies on retraction concentrated on the reasons for retraction. Fang et al. (2012) studied a large set of more than 2000 retracted articles indexed by PubMed and found that more than 67% of the retractions are due to misconduct, including fraud and suspected fraud. Steen (2011a) also studied a subset of biomedical, retracted articles retrieved from PubMed, and contrary to Fang et al. (2012), he found that error was the most common reason for retraction. Another study by Wager and Williams (2011) was also based on biomedical retracted articles, and like Steen (2011a) found that error was most prevalent. Temporal aspects were also studied, for example by Fanelli (2013) and Steen et al. (2013). A review article on scientific misconduct was recently published (Gross 2016).

The continued positive citations of retracted articles are a serious issue that warrants a closer examination. As can be seen, most of the previous studies concentrated on biomedical research. Our approach was different, as we retrieved in October 2014 retracted papers from a major scientific publisher, Elsevier, thus our sample includes papers from all areas of science and social science. We selected 15 retracted articles, according to the following criteria: retracted between 1995 and 2014 that received the highest number of citations between January 2015 and March 2016 (called recent citations in this paper) that occurred definitely post retraction, even when the retraction date of the article is not specified. By conducting a context analysis of each of the citations they received, we sought out to find whether they are negatively, positively or neutrally mentioned.

## Data collection

ScienceDirect, Elsevier’s full text database was accessed in October 2014. The database was queried for the term “RETRACTED” in the article title and its retraction notice. In ScienceDirect, each retracted article is preceded with the word “RETRACTED”. In addition, each Elsevier journal incorporates a retraction notice which explains who retracted article and the reason for retraction. This allowed us to manually code each article in our dataset with an additional field “retracted by” that represented the person/s requesting the retraction. An alternative search strategy is to search by Document type: Erratum for retract*, which retrieves retraction notices, however less results were retrieved, because not all retractions are accompanied by separate retraction notices. A sample of the results from the alternative strategy was checked against the results of the search strategy applied in this study: all articles in the sample were found in the data set created by our search strategy.

A total of 1203 results retrieved from which 987 were retracted articles. The results excluded were retraction notices, duplicates and papers whose original titles included the word “retracted”.

**Fig. 1:**
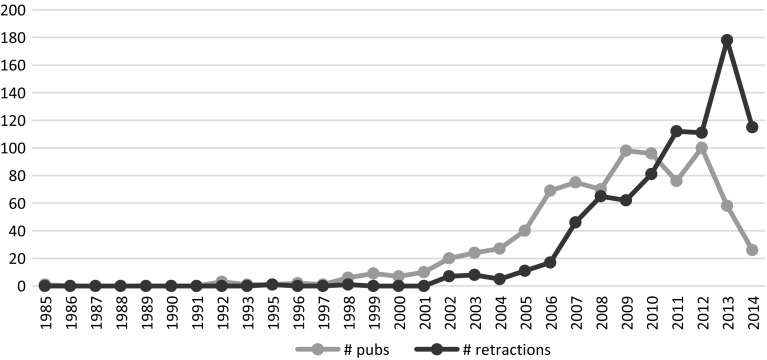
Number of publications per year that were later retracted and the number of publications retracted by year

**Fig. 2:**
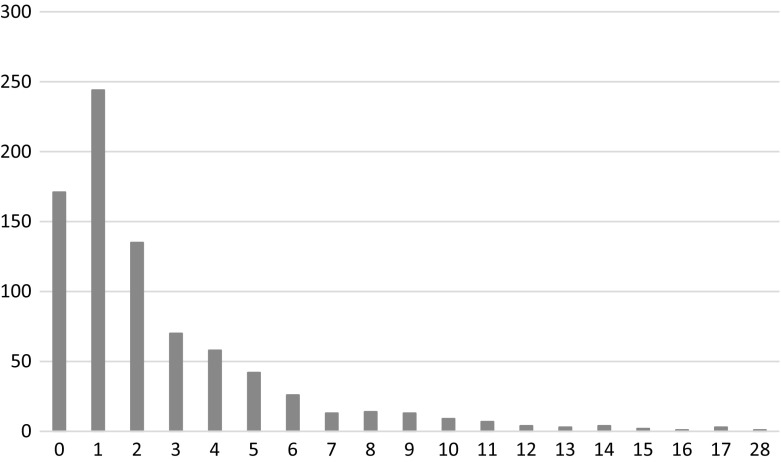
Number of years between publication and retraction

All articles had retraction notices, but the date of retraction was not available for all of them, out of the 987 retracted articles, retraction date was available for 820 articles (83.10%). Elsevier acknowledged that retraction dates are not available for all articles. However, since data were collected on October 2014, and citation that appears from 2015 onwards is definitely a post retraction citation. The distribution of the publication and the retraction years of the 820 retracted articles with known retraction year is visualized in Fig. 1. We see that there is a time lag between the publications and the citation. The range of the time lag for our sample of retracted documents is between 0 and 28 years (Fig. 2). The average time for retractions is 2.5 years. The drop in the number of retracted papers in 2013 and 2014 is most probably temporary, as retractions take time.

For the current paper we chose all retracted articles that were cited more than ten times between January 2015 and March 2016. Fifteen such articles were identified. These 15 articles received altogether 267 citations between January 2015 and March 2016. We were unable to access 29 citing papers (mainly book chapters, or articles in Chinese), thus the analysis relies on 238 citing documents.

Each citing document was inspected to identify the precise mention of the retracted article within the text. Each mention was categorized as follows:

1. *Positive* A positive citation indicates that the retracted article was cited as legitimate prior work and its findings used to corroborate the author/s current study.
2. *Negative* A negative citations indicates that the authors mentioned the retracted article as such and its findings inappropriate.
3. *Neutral* A neutral citation indicates that the retracted article was mentioned as a publication that appears in the literature and does not include judgement on its validity.

## Findings

### **Case study 1**

Donmez, G., Wang, D., Cohen, D. E., and Guarente, L. (2010). RETRACTED: SIRT1 Suppresses β-Amyloid Production by Activating the α-Secretase Gene ADAM10. Cell, 142(2), 320–332.

**Fig. 3:**
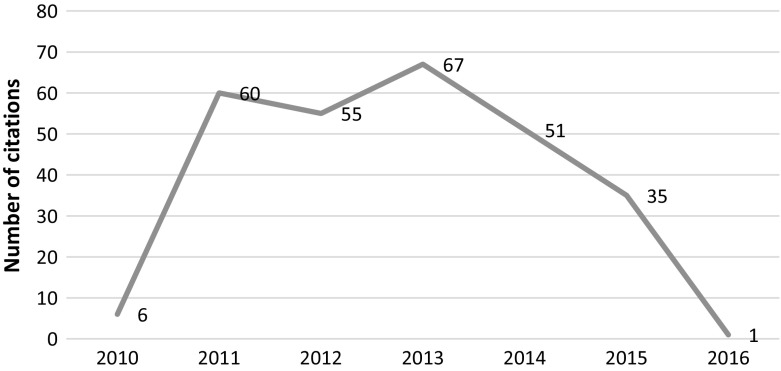
Number of citations per year—Donmez et al. article

This article was published in 2010 in Cell and retracted in 2014 due to irregularities in graphs and data misrepresentation in the images. The article was retracted at the request of the authors, who stated *“…the level of care in figure preparation in Donmez* et al*. falls well below the standard that we expect, and we are therefore retracting the paper”* but added that “*We believe that these errors do not affect the conclusions of experiments in the paper”.* The article was cited 275 times since its publication with most recent citations tracked in 2015 and 2016, clearly post-retraction (see Fig. 3).

**Fig. 4:**
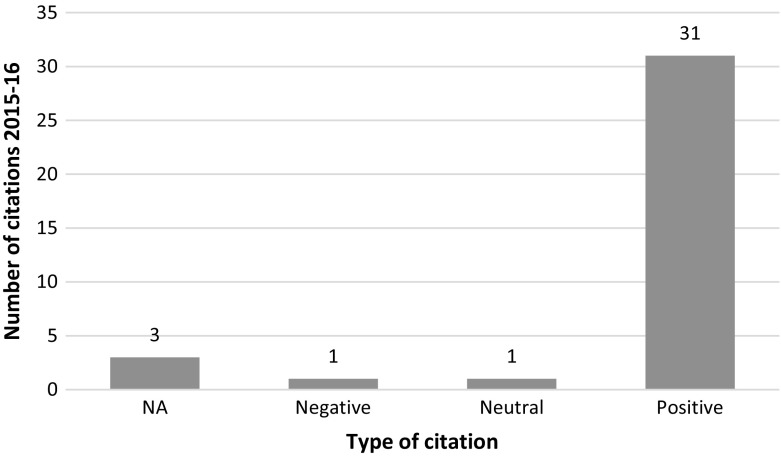
Distribution of the post retraction citations of the Donmez et al. article

We conducted an individual content analysis of the most recent 36 citations which were tracked in 2015 and 2016. We were able to analyze 33 citing articles in context. Our results show that the citations are mostly positive (see Fig. 4). One negative mention was found in a letter to the editor of *the Journal of Korean Medical Science* giving the above article as an example of how altered graphics are causing bias in the biomedical field and result in numerous articles being retracted (Seifirad et al. 2015). One of the reasons for continued citations could be that retraction notice indicated that the conclusions of the study were not influenced by the image manipulation. See PubPeer (2013) or Oransky (2014a) for further details.

### **Case 2**

Séralini, G. E., Clair, E., Mesnage, R., Gress, S., Defarge, N., Malatesta, M.,… and De Vendômois, J. S. (2012). RETRACTED: Long term toxicity of a Roundup herbicide and a Roundup-tolerant genetically modified maize. Food and Chemical Toxicology, 50(11), 4221–4231.

This article, published in 2012 was the subject of a debate surrounding the validity of the findings, use of animals and even accusations of fraud. Its publication and retraction process have resulted in the “Séralini affair” which became a big media news item (Séralini affair 2016). The article described a 2-year study of rats which were fed genetically modified (GM) crops and showed increased tumors. The study, which was also scrutinized by government agencies, received major media attention that resulted in the creation of a social movement against GM food. Despite the accusation of fraud and fabrication of results, the editors found no such evidence to that effect. However, the article was retracted in 2013 because of the *“low number of animals”* used in this study which lead to the conclusion that *“no definitive conclusions can be reached with this small sample size”.*

**Fig. 5:**
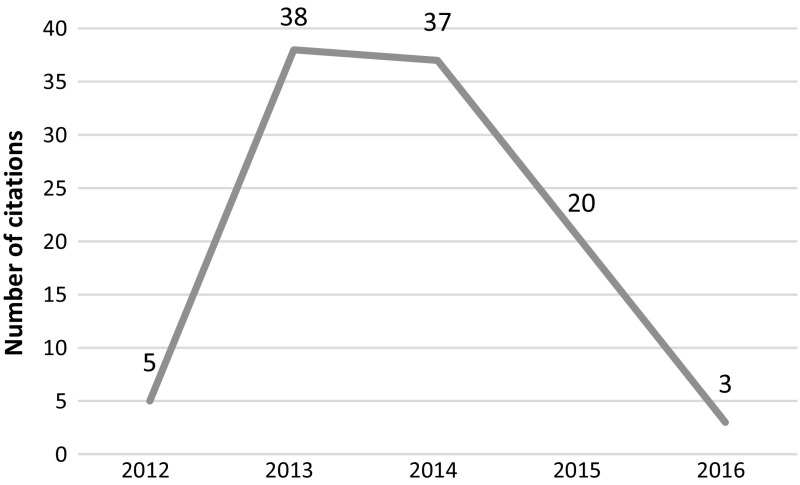
Number of citations per year—Séralini et al. article

This article was cited 103 times since its publication in 2012 (see Fig. 5) with 60 citations after retraction, out of which 24 citations occurred recently (2015–2016). We were able to access 23 out of the 24 articles. Post-retraction citations are divided. Although more citations are seen to be negative (9 out of 23; 39%), the positive (6, 26%) and neutral ones (7, 31%) are also present. The negative citations mostly point to the media frenzy around the results. Positive mentions appear in similar studies which claim that concerns raised by the GM study are valid and the dangers of GM foods to humans should be studied further.

The study was republished in 2014 by *Environmental Sciences Europe*. The republication of the study stirred another controversial discussion in the scientific community with several scientists writing letters expressing their concerns regarding the appearance of the same study in another journal. For further details, consult Oransky (2013).

The republished article received 17 citations in 2015 and 2016. The vast majority of them being positive mentions (87%), however some criticism towards the peer-review practices of the retracting editors were also detected (Loening, 2015). The one negative mention of the re-published article was criticism towards the media frenzy around the topic and the inability of the scientific community to refute invalid results. The authors state that *“Although scientists have investigated each GMO crisis and reached scientific and rational conclusions, they have less ability to disseminate information than the media, so the public is not promptly informed of their rational and objective viewpoints as experts* (Xia et al. 2015).

### **Case 3**

Mukherjee, S., Lekli, I., Gurusamy, N., Bertelli, A. A., and Das, D. K. (2009). RETRACTED: Expression of the longevity proteins by both red and white wines and their cardioprotective components, resveratrol, tyrosol, and hydroxytyrosol. Free Radical Biology and Medicine, 46(5), 573–578.

**Fig. 6:**
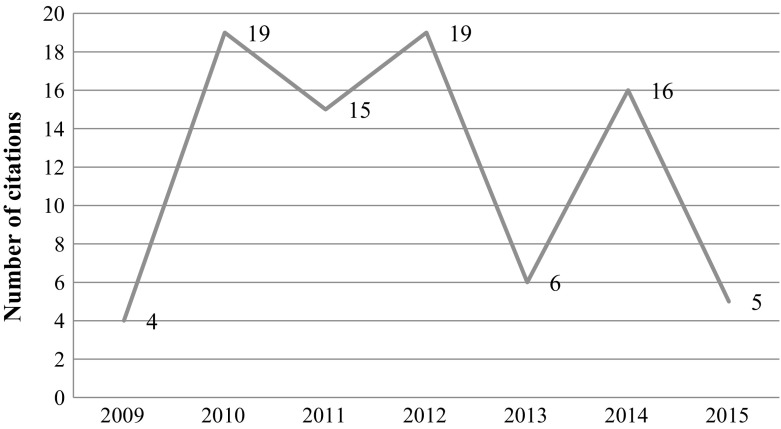
Number of citations per year—Mukherjeee et al. article

The leading author of the paper, Prof. Dipak Das and his lab at the University of Connecticut Health Sciences Center were the subject of an ethical investigation by the university. The results of the university’s investigation led to the retraction of all of Dr. Das papers due to scientific misconduct and data manipulation—up to 20 retractions according to Retraction Watch (Oransky 2014b). This particular paper was investigated by the journal’s ethics committee along with an additional paper that appeared in the same journal. The journal’s ethics committee “*analyzed the data presented, and then further concluded that …. on re*-*examination of these two FRBM (Free Radical Biology and Medicine) papers that they contain clear evidence of obvious cutting, pasting and manipulation of data in experimental blots*.” The article, which was retracted in 2012, received 85 citations since its publication in 2009, 21 of which occurred in 2014 through 2015 (see Fig. 6).

For this paper we were able to access 17 out of the 21 recent citations. All of these quoted the article’s findings as legitimate. For example, *“Plants containing resveratrol, a potent antioxidant, has been used widely in the treatment of various ailments”* (Pangeni et al. 2014) or “*Recent studies have also shown that red wine upregulates the protein expression of sirtuin”* (Romain et al. 2014).

### **Case 4**

Walumbwa, F. O., Wang, P., Wang, H., Schaubroeck, J., and Avolio, B. J. (2010). RETRACTED: Psychological processes linking authentic leadership to follower behaviors. Leadership Quarterly, 21(5), 901–914.

**Fig. 7:**
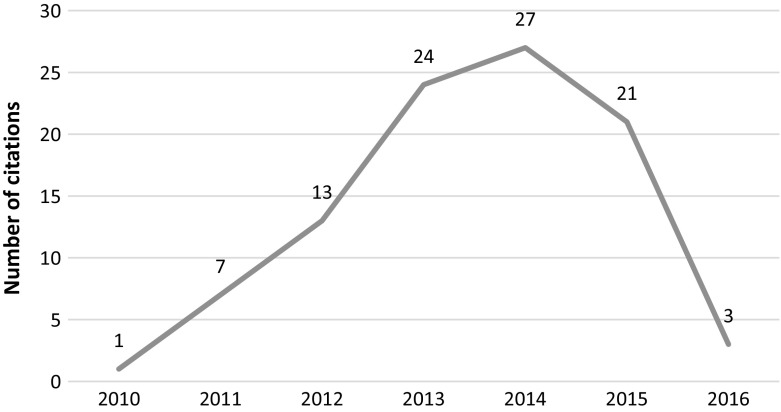
Number of citations per year—Walumba et al. article

This article was retracted in 2014 due to serious data manipulation and falsification. In the retraction notice, the editors of the journal went to great lengths to examine and re-examine the statistical claims made by the authors using the services of 3 separate methodologists. Following the methodologists’ findings of irregularities in the reported data and falsification of results, and the authors’ lack of proper response to their findings, the article was retracted from the journal. However, the article continued to be cited despite the lengthy and detailed retraction notice (see Fig. 7). It should be noted that this article was retracted by the editors of Leadership Quarterly together with four other articles of Fred O. Walumbwa (Retraction Watch 2014).

A close examination of the post retraction citations (2015–2016) shows that all 23 citations were positive citations (we unable to access one citing document), meaning that the citing authors used findings from this article to support their findings. The subject of “authentic leadership” is popular in management studies and has seen a surge in publications since 2012. This could explain the overall positive citations of the article.

### **Case 5**

Li, C., Tao, X. M., and Choy, C. L. (1999). RETRACTED: On the microstructure of three-dimensional braided performs. Composites Science and Technology, 59(3), 391–404.

**Fig. 8:**
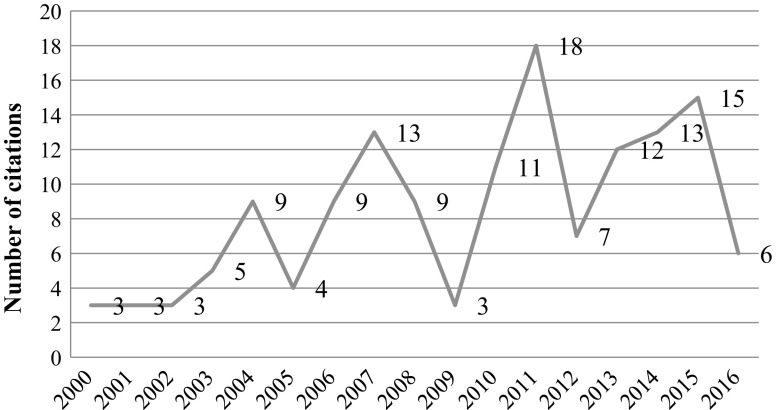
Number of citations—Li et al. article

This article, published in 1999 was retracted due to an identical version which was published in 1997. In the retraction notice the editors state that “*The article duplicates significant parts of a paper that had already appeared in* [*J China Textil Univ, 1997, 14*(*3*)*, 8*–*13*]”. The authors in this case re-used data they already published on and re-published it in a different journal. However, this article has been cited even in recent years despite being retracted for many years (see Fig. 8). A content analysis of the 21 recently citing articles from 2015 and 2016 shows that this article is being referred to mostly in positive context or mentioned as a legitimate piece in the literature (all 18 accessible citing articles were either positive (11) or neutral (7)). The retraction notice of this article is not dated, however Elsevier informed us that the paper was retracted in 2013. A possible reason for continued citations, is that the article published in the *Journal of China Textile University* is not accessible.

### **Case 6**

Hwang, Eun-Sun, and Gun-Hee Kim. (2007). RETRACTED: Biomarkers for oxidative stress status of DNA, lipids, and proteins in vitro and in vivo cancer research. Toxicology 229 (1–2), 1–10.

**Fig. 9:**
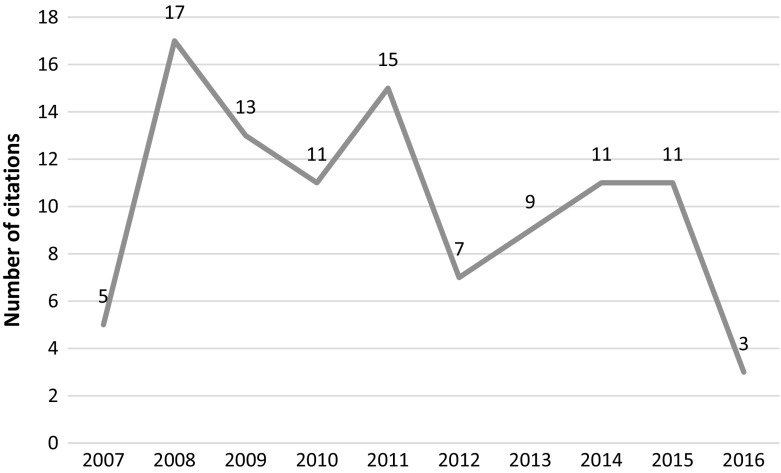
Number of citations—Hwang et al. article

This article, published in 2007 reports on the use of biomarkers to measure oxidative stress status has on the body and which might cause diseases such as cancer. The article was retracted due to plagiarism whereas the author copied complete sentences from a previously published paper without citing it. This article received over a hundred citations since its retraction in 2007, even though it was retracted already in 2007, only ten months after the article first appeared, with 13 recent citations in 2015 and 2016 (see Fig. 9). Examining the latest citations from 2015 and 2016, all the citations accessible to us (11 publications) were positive.

It should be noted that the plagiarized article (Mayne, 2003) was cited 304 times, out of which 24 occurred in 2015–2016. None of the 14 recent citing papers of the retracted article cited the article authored by Mayne.

### **Case 7**

Qiang, L., Fujita, R., Yamashita, T., Angulo, S., Rhinn, H., Rhee, D., and Abeliovich, A. (2011). RETRACTED: Directed conversion of Alzheimer’s disease patient skin fibroblasts into functional neurons. Cell, 146(3), 359–371.

**Fig. 10:**
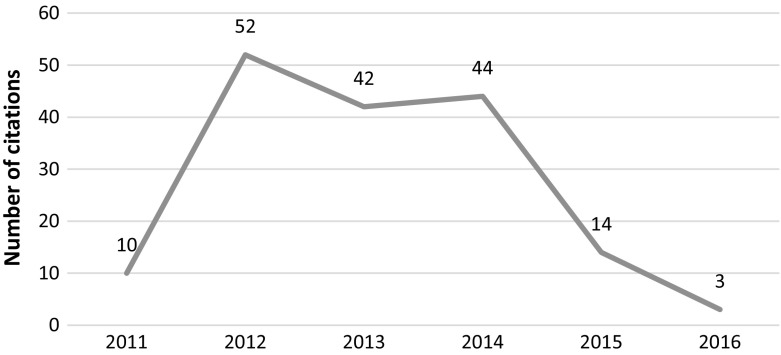
Number of citations per year—Qiang et al. article

This article, published in 2011 was retracted in 2014. The reason for retraction was misconduct by one of the authors who admitted to *“inappropriately manipulating image panels and data points, as well as misrepresenting the number of repeats performed, in the experiments presented” (Retraction notice).* This type of retraction has a direct implication on the validity of the findings and the research overall. Despite of the problematic issues with the study, it has been cited and still receives citations in 2016 (see Fig. 10). Out of the 17 recent citations, 12 are positive two are neutral, two inaccessible and one negative. The negative citation is a letter to the editor of the journal discussing the misconduct of the authors.

### **Case 8**

Ji, Z. X., Sun, Q. S., and Xia, D. S. (2011). RETRACTED: A framework with modified fast FCM for brain MR images segmentation. Pattern Recognition, 44(5), 999–1013.

**Fig. 11:**
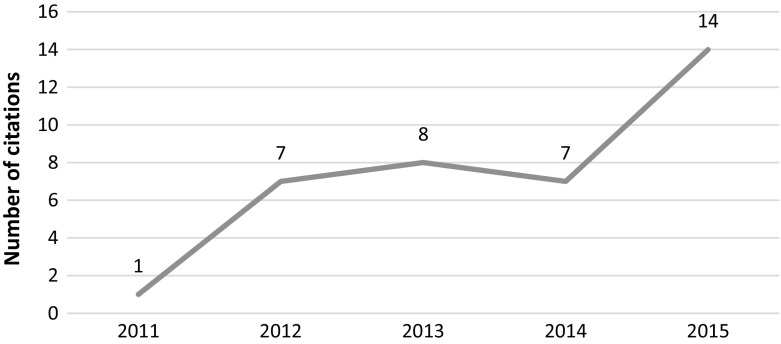
Number of citations per year—Ji et al. article

This article was published in 2011 and retracted in 2014. The reason for the retraction was its significant similarity to an article published two years earlier in a conference proceedings by other authors. Interestingly the retraction was at the request of the authors. Most of the citations to this article were positive. This article received 37 citations over the years (See Fig. 11), while the original paper (Li et al. 2009) has received 38 citations. None of the articles citing the retracted article cited the original article as well. Interesting to note the rise in the number of citations after the retraction (14 citations). The single negative citation in this case, is not negative in the sense that it flags the cited article as retracted; it simply states that the method of the citing article is superior to the method in the retracted article.

### **Case 9**

Zhao, R., Zhang, Z., Song, Y., Wang, D., Qi, J., and Wen, S. (2011). RETRACTED: Implication of phosphatidylinositol-3 kinase/Akt/glycogen synthase kinase-3β pathway in ginsenoside Rb1’s attenuation of beta-amyloid-induced neurotoxicity and tau phosphorylation. Journal of Ethnopharmacology, 133(3), 1109–1116.

**Fig. 12:**
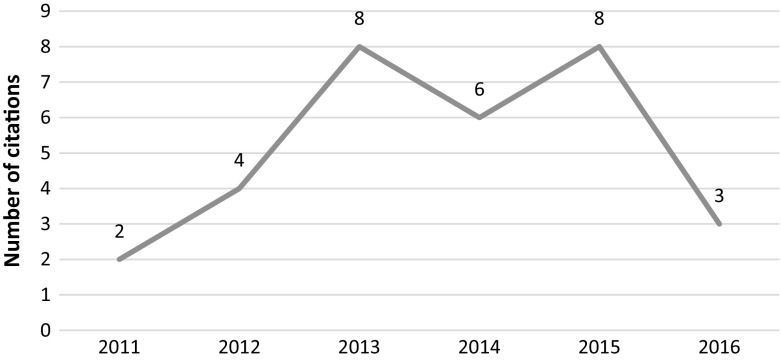
Number of citations per year—Zhang et al. paper

This article was retracted in 2012 due to misconduct on the part of the authors. The retraction notice states that “*The authors have duplicated content as well as misleadingly modified figures that had already appeared” (retraction notice).* The authors published three versions of the same paper. One of the two other versions was also retracted. The retraction notice implies that the authors not only plagiarized their article but also engaged in data manipulation which has severe bearing on the validity of the study’s results. However, this article is seen to be cited recently (see Fig. 12) despite of the profound reasons which led to its retraction. The article has been cited 28 times, with eleven recent citations, the other retracted article has been cited seven times with two recent citations, while the non-retracted version has been cited 23 times with 8 recent citations. Thus, the retracted article in the Journal of Ethnopharmacology is more cited both pre-and post-retraction than the non-retracted version. All three journals have similar impact factors (between 3.1 and 2.5 for 2015).

In addition, all the recent citations accessible to us (10 papers out of the 11 recent citing publications) were positive, citing the findings of the retracted paper as valid. These citations appear after a retraction notice has been issued and the reasons made known to the scientific community.

### **Case 10**

Nanjawade, B. K., Manvi, F. V., and Manjappa, A. S. (2007). RETRACTED: In situ-forming hydrogels for sustained ophthalmic drug delivery. Journal of Controlled Release, 122(2), 119–134.

**Fig. 13:**
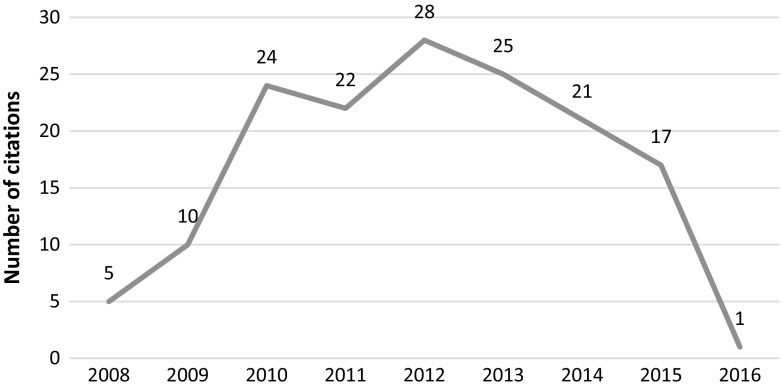
Number of citations per year—Nanjawade et al. article

In this case, the article was published in 2007 and retracted in 2013 due to plagiarism. The retraction notice states that the authors have plagiarized parts of a large number of previously published papers by other authors. Yet, as can be seen in Fig. 13 the article was highly cited well after a retraction notice was issued. The citations (15 accessible out of the 18 citations in 2015–2016) were all positive citing the article’s findings as valid and the authors as legitimate owners of the research.

### **Case 11**

Yamagata, K., Fujiyama, S., Ito, S., Ueda, T., Murata, T., Naitou, M., and Kato, S. (2009). RETRACTED: Maturation of MicroRNA is hormonally regulated by a nuclear receptor. Molecular Cell, 36(2), 340–347.

**Fig. 14:**
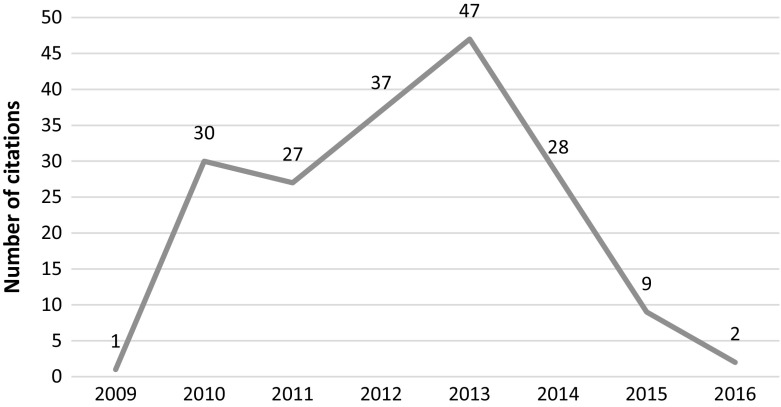
Number of citations per year—Yamagata et al. article

This article poses an interesting case. It was published in 2009 and was retracted in 2014 by request from the authors. They were using an external laboratory to conduct some of the experiments and discovered that the laboratory mishandled the materials and manipulated the images thus undermining the authors confidence in the results. Even though the article was retracted in 2014 it is still cited through 2016 (see Fig. 14). Our analysis showed that all of the accessible recent citations (10) listed in Scopus the citations were positive. One item, a book was not accessible. This is surprising in light of the fact that the results of the study are practically invalid due to the faulty experiments. One should note that the last author, Shigeaki Kato, had 25 retracted papers in 2014 (Oransky 2014c) and this number rose to 38 by April 2016 (Palus, 2016). As one of the readers Palus’ post commented: “Wow. he’s had more papers retracted than I’ve had published (by far!)”, however this is still less than 10% of Kato’s publications indexed by Scopus (476), with an h-index of 85. This retracted article is part of his h-core, as it received so far more than 175 citations.

### **Case 12**

Vaidyanathan, R., Kalishwaralal, K., Gopalram, S., and Gurunathan, S. (2009). RETRACTED: Nanosilver—The burgeoning therapeutic molecule and its green synthesis. Biotechnology Advances, 27(6), 924–937.

**Fig. 15:**
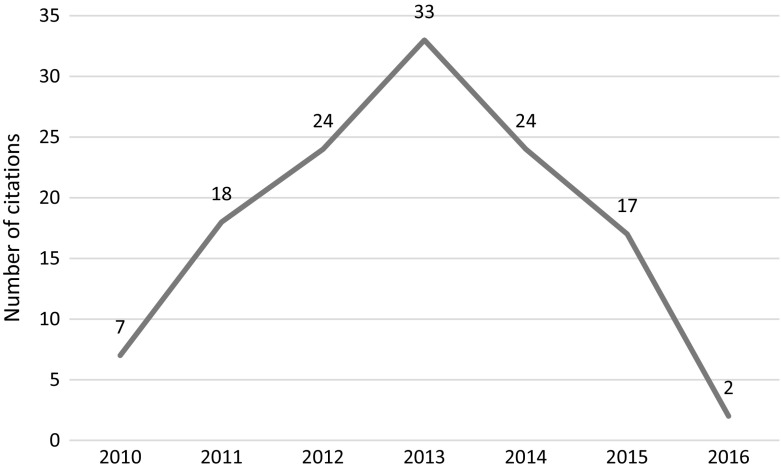
Number of citations per year—Vaidvanathan et al. article

The reason for the retraction of this article is plagiarism. The paper was retracted in 2010. The authors compiled this paper by using large parts of previously published papers. The retraction notice lists nine different papers from which the authors copied and used to construct the article. Out of the 19 recent citations 17 were positive and 2 neutral, and there is no mention of this article as plagiarized in any context. Note that 118 out of the 125 citations the paper received until March 2016 are post-retraction citations (see Fig. 15).

### **Case 13**

Liu, X., Liu, H., Wang, S., Zhang, L., and Cheng, H. (2006). RETRACTED: Preparation and thermal properties of form stable paraffin phase change material encapsulation. Energy Conversion and Management, 47(15), 2515–2522.

**Fig. 16:**
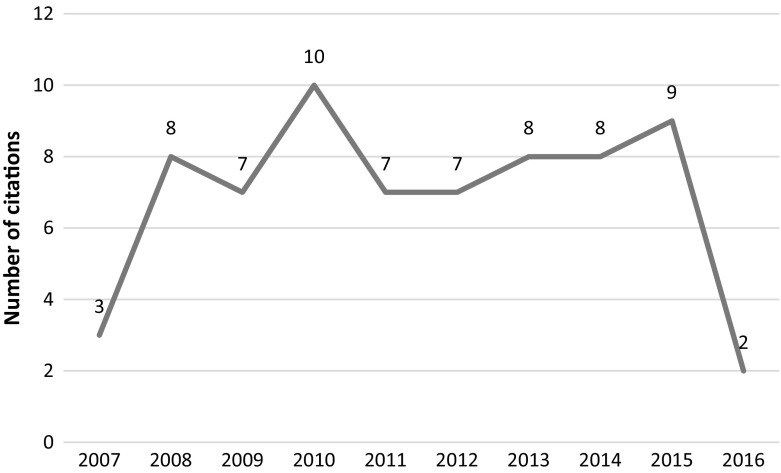
Number of citations per year—Liu et al. article

This article presents another form of plagiarism where the authors reused their previously published paper to compile a new article. The plagiarized article published in the same year in a different journal with an identical title was also retracted. Neither of the articles have a recorded retraction date, however Elsevier informed us that the articles were retracted in 2009. The retraction was initiated by the editor who states that there are significant parts which already appeared elsewhere. The article continues to be cited even in 2016 (see Fig. 16). Out of the 11 recent citations, 10 were accessible and all 10 were positive. In this case too, the number of post-retraction citations is considerably larger than the number of pre-retraction citations.

### **Case 14**

Nabae, Y., Moriya, S., Matsubayashi, K., Lyth, S. M., Malon, M., Wu, L.,… and Miyata, S. (2010). RETRACTED: The role of Fe species in the pyrolysis of Fe phthalocyanine and phenolic resin for preparation of carbon-based cathode catalysts. Carbon, 48(9), 2613–2624.

**Fig. 17:**
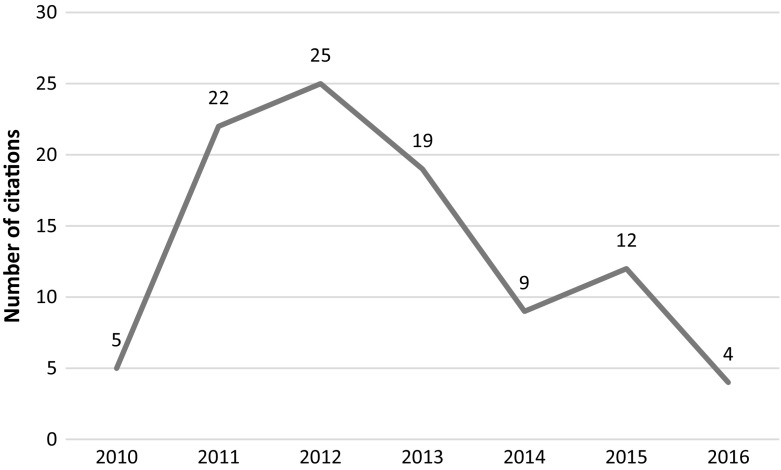
Number of citations by year—Nabae et al. article

This paper was retracted in 2012, due to the discovery that one of the author manufactured false data to support its findings. The retraction statement states that the case was investigated by the institution which claimed that the data are correct. Yet, despite of the fact checking performed by the institution, the co-authors of the paper requested that it will be retracted. The co-authors name one of the authors as the responsible party for the false data used. As can be seen from Fig. 17, the article continues to be cited through recent years despite the profound doubt surrounding the validity of its results. All recent accessible citations (14 out of 16) were positive or neutral. Further details on this retraction are reported by RetractionWatch (2013).

### **Case 15**

Liu, X. F. (2014). RETRACTED: Substitution reactions of diiron dithiolate complexes with phosphine or isocyanide ligands. Journal of Organometallic Chemistry, 750, 117–124.

This article was retracted in 2014, due to plagiarism which not only pertained to the text but also to the methodology presented. The editor in chief states in the retraction notice that the *“some of the work reported as new in this paper, was previously conducted by someone else… the…method used and the proposed mechanism… are similar to those previously reported [previously by someone else] and …portions of the manuscript are worded identically to those in manuscripts that have been published”* (retraction notice). As can be seen from the statement, this article presents deep and compound case of plagiarism that ranges from text to methods. Yet it is still positively cited, with 12 citations in 2015: 9 positive and 3 neutral. Since the article was retracted in the same year as it was published, it is difficult to decide what portion of the ten citations received in 2014 were pre-retraction citations. It should be noted that 8 of the 12 recent citations are from a single author, Wei Gao.

## Discussion and conclusions

As can be seen from the examples above, retracted articles continue to be cited years after retraction and despite retraction notices being posted on publishers’ platforms. There are different reasons for retraction some articles were retracted for ethical reasons (plagiarism, self-plagiarism or publishing multiple versions of the same paper)—8 out of the 15 studied retractions belong to this category. Here the problem is not with the validity of the findings, but in addition to the ethical issue, the authors of the plagiarized papers are deprived of citations that go to plagiarizing paper.

More serious are the cases where the data or the images were manipulated. This happened in 8 out of the 15 cases studied (Case #9: the article was both self-plagiarized and some of the images were manipulated). Manipulated data lead to unreliable conclusions, which might have far reaching implications (e.g. whether genetically modified corn causes cancer) especially when these articles are continued to be cited after retraction, without stating that the article has been retracted.

Endeavoring to understand the motives behind post-retraction citations is difficult. However, we propose a few possible explanations for post-retraction citations:

1. The full text of retracted articles is freely available to all

All the retracted articles in our case study were published in journals behind a paywall. When an article is retracted, it becomes freely accessible for all on the publisher’s website with a RETRACTED stamp on it. The ease of access to retracted articles could be a reason for using and citing them. It is quite plausible that copies without the RETRACTED stamp can be located on the Web, and the authors of the citing article are not even aware that the cited article has been retracted.

2. Public and/or Media Attention

In some cases, post-retraction citations could be the result of public and/or media attention. For example, the Séralini (Case #2), article evoked a public debate regarding the safety of genetically modified (GM) foods. This debate continued over media channels well after the article was retracted resulted in a call to enforce labelling all GM food items. This type of public attention could explain the continuing interest in the study despite some of the methodological problems found by the editor. In addition, the article was republished and thus continues to be cited despite the fact that the authors did not modify the original version. It seems that public or media attention can cause a rise in the number of negative citations (Fagan et al. 2015; Nau 2013).

In the case of the Mukherjee (Case #3), article, again, public and media interest could explain its continuing citations. Resveratrol was hailed by the media as an important supplement that could ensure longevity and good health. Today, Resveratrol is offered as an off the counter supplement available in vitamin shops. This is an indication that the study’s results were accepted despite of the inconclusiveness of the results and the problematic study design.

Similarly, Walumbwa, (Case #4), sparked media attention offering catchy corporate leadership concepts. Terms such as ‘*authentic leadership’* and ‘*followers’ dynamic’* became popular topic of media and business management interest. These concepts became the topics of several management articles and books (e.g., Edú-Valsania et al. 2016; Gatling et al. 2016; Van Bogaert 2016).

3. Data and image manipulations are ignored

In other cases, the retraction notice is not clear enough or indicates that the manipulations do not affect the validity of the findings. For example, the Donmez (Case #1), article was retracted because of poor graphing and data representation which is a serious issue in the biomedical sciences. In spite of this, the retraction notice states that these faults do not apply to the validity of its results which could explain the continuing post-retraction citations of the article.

4. Retraction due to self-plagiarism or duplicate publication

Post-retraction citations of articles that were flagged for self-plagiarism are also common. Liu et al. (Case #13) were accused of re-using their own data and large sections of articles they published before. These practices violate the principle of originality in science whereas each published work must be original and not published anywhere else. However, neither the data nor the findings were challenged by the editors which make the study valid despite of it being a duplicate of previously published articles.

Nonetheless, our analysis shows that there are many instances where post-retraction citations are seen to articles that were retracted due to methodological flaws, data fabrications and other reasons that make the articles and their findings invalid. This phenomenon is the most concerning. When such articles are referred to and their results are listed in the text as valid step stones in science and discovery, the integrity and advancement of the scientific endeavor is jeopardized.

In this study, we not only looked at the citation distribution pre and post-retraction, but examined all recent citations that were definitely post-retraction, i.e. were published after we collected the retracted articles from the publisher. Only a few studies examined post-retraction citations and their sentiment (positive, neutral or negative) and these studies were conducted mainly in the medical field (e.g., Garfield and Welljams-Dorof 1990; Kochan and Budd 1992; Budd et al. 1999; Steen 2011b).

To sum up our quantitative findings, out of the 238 post retraction citations analyzed, 198 (83%) were positive, 28 neutral (12%) and only 12 (5%) negative. Only in one case, the Séralini paper (Case #2) there were more negative than positive citations. These percentages are quite similar to those in (Kochan and Budd 1992). In addition, the number of citations these articles received between January 2015 and March 2016 is considerably higher than the average number of citations for articles in the same journal and publication year, except for the papers that appeared in Cell journals (Cases #1, 7, 11). It should be noted that in the reference lists of the citing papers the retracted papers are almost never flagged as such. This issue was also mentioned by Bornemann-Cimenti et al. (2015) and by Neale et al. (2010). There are limitations to our findings as we considered a small specific sample, and thus it is not possible to generalize, but still in light of our findings, we recommend the following:

1. Publishers should conduct thorough reference checks to detect citations of retracted articles and remove them. If an article lists or refers to a retracted publication, a clear notice of retraction should be listed in the reference list and the reference text as well. Editors should question why authors cite retracted publications and unless the editor and the peer reviewers are convinced that the citation is essential, references to retracted articles should be removed.
2. The current practice of stamped retracted articles freely available should be reconsidered. The full text of the retracted article should not be freely available on platforms such as ScienceDirect or others. Although versions of these articles may appear elsewhere, the journal websites should not carry these versions and make it difficult for authors to download, read and consequently cite retracted articles. It is rather puzzling why retracted articles are freely available, while the huge majority of the commercial publishers’ articles are behind a paywall.
3. Publishers should closely collaborate with content aggregators and create a workflow where each retraction notice can be seen on all platforms. There were quite a few instances where we observed a retraction notice on the publisher platform with no parallel notice in content aggregators such as PubMed. In these cases, researchers who use only PubMed for example might think that the article that they are referring to is valid.
4. Although COPE (Committee on Publication Ethics) provides guidelines for editors on how to handle retractions (COPE 2015; Wager et al. 2009), there are no guidelines on what the editors and the publishers do when they notice references to retracted articles. COPE has more than 10,000 members (COPE 2016), and we believe it should provide guidelines also for handling post-retraction citations. Such guidelines might include recommendations such as clearly tagging retracted articles in the reference list or asking to remove such references altogether or ask the authors for clear explanation why the retracted paper is referenced.
5. In order to ease the identification of references to retracted articles during the peer review process, a database of retracted articles including the reasons for the retraction should be set up. As one of the reviewers of the article pointed out there are tools in some editorial systems that flag notices linked to the cited article on PubMed.

## Further study

We conducted a case study based on 15 retracted articles. This is obviously not enough; further larger scale studies are needed to support the current findings. Most previous studies on retractions concentrated on the biomedical field and drew their data from PubMed. Here, we looked at retracted articles from all Elsevier journals, in our sample, 6 out of the 15 articles were not indexed by PubMed. Additional aspects should be explored, e.g. retraction notices that appear only on publishers’ platforms but not on content aggregation platforms as opposed to those who appear in both. This could be a factor in the amount of citations these articles receive. In this study, we noticed instances where articles were flagged as retracted on the publisher’s platform but not on content aggregators’ platform. This could contribute to the post-citations phenomenon as authors are not aware of the retraction notice because they used a database that was not updated. A close study into this could assist with creating some clear guidelines for publishers and content aggregators to streamline the process of flagging and removing flawed studies. Another issue to be examined is the comparison of the post retraction citation rates of retracted articles with the citation rates of articles in the same journal issue that were not retracted along the lines of the previous studies (Furman et al. 2012; Neale et al. 2010).
